# Serum from mice immunized in the context of Treg inhibition identifies DEK as a neuroblastoma tumor antigen

**DOI:** 10.1186/1471-2172-8-4

**Published:** 2007-03-30

**Authors:** Jin Zheng, M Eric Kohler, Qingrong Chen, James Weber, Javed Khan, Bryon D Johnson, Rimas J Orentas

**Affiliations:** 1 Department of Pediatrics, Medical College of Wisconsin and the Children's Research Institute, Children's Hospital of Wisconsin. 8701 Watertown Plank Rd. Milwaukee, WI 53226, USA; 2 National Cancer Institute, Pediatric Oncology Branch, Oncogenomics Section, U.S. National Institutes of Health, Advanced Technology Center, 8717 Grovemont Circle. Gaithersburg, MD 20877, USA

## Abstract

**Background:**

We have developed a cell-based vaccine that features the expression of both CD80 and CD86 on the surface of a murine neuroblastoma cell line. The cellular immunity induced by this vaccine is enhanced by treatment with antibody that interferes with T-regulatory cell (Treg) function and we report here that immunization combined with interfering with Treg function also produces a profound serological effect. Serum from mice immunized with our cell-based vaccine in the context of Treg blockade was used to screen a cDNA expression library constructed from the parental neuroblastoma tumor cell line, AGN2a.

**Results:**

Serum from mice vaccinated in the context of Treg blockade identified a number of potentially oncogenic transcripts that may serve as important immune targets in a tumor-derived cDNA library screen. This novel approach identified far more candidates than could be seen with serum derived from vaccine-treated only, Treg-depleted only, or tumor-bearing mice. The most commonly identified tumor-associated antigen, using serum from immunized and Treg-depleted mice, was the DEK oncogene. Altered expression of the DEK oncogene has been implicated in a number of human cancers. Importantly, we were able to demonstrate that the DEK oncogene also induces a T cell response.

**Conclusion:**

The use of post-vaccine immune serum in this report differs from previous approaches where serum collected at the time of cancer onset or diagnosis and was used for tumor antigen identification. We hypothesize that the use of diagnostic serum samples may be inadequate for the clinical translation of this approach, and that identification of protective immunogenic tumor antigens may require the use of serum from post-treatment or vaccinated subjects. The identification of DEK as a tumor-associated antigen capable of eliciting a T cell response validates our experimental approach and argues for the antigens we have identified here to be evaluated as targets of effector immunity and as vaccine candidates.

## Background

Advanced neuroblastoma poses a grave clinical challenge and still awaits effective therapy. Early clinical observations, combined with a slight but demonstrable positive impact of bone marrow transplantation on outcome has motivated the development of immune approaches to therapy [1-4]. In murine models of human neuroblastoma, anti-tumor immunity can be generated using cell-based vaccines where tumor cells have been genetically modified to express soluble cytokines or cell-surface immunostimulatory molecules [5-7]. Our own work has demonstrated that cancer cell-based vaccines expressing multiple immune co-stimulatory molecules in the murine neuroblastoma cell line AGN2a can transform this tumor cell line in to a vaccine that induces strong cell-based immunity to the unmodified parental cell line [8,9]. Based on the ability to induce an immune response with cancer cell-based vaccines, human trials with neuroblastoma patients have been carried out [10]. Although these cell-based cancer vaccines did not prove immediately effective, they were demonstrated to be safe and are ripe for further optimization [11].

In experimental systems, immunity to neuroblastoma can be amplified by the blockade of T-regulatory cell (Treg) function with anti-CD25 antibody (B.D. Johnson, et al., 2007, J. Immunother., in press). Treg are known to suppress the immune response to self-antigens, including tumor-self antigens, and thwarting this tolerogenic role by their depletion has become a major focus in the development of new immunotherapeutic strategies to treat human malignancy [12,13]. Golgher et al. have demonstrated that CD25+ T cell depletion uncovers immune responses to the tumor cell type used as a vaccine, and importantly that this response broadens to include other syngeneic tumor cell types [14]. Given the ability to induce immune recognition of what are normally considered "self" antigens upon Treg blockade, we reasoned that treatment of experimental animals with cell-based cancer vaccines in the context of anti-CD25 antibody treatment would induce a strong anti-neuroblastoma immune response. The proposed use of serology to uncover T cell antigens is supported by the recent description of antibody as well as T cell responses to the DBY minor histocompatibility antigen in allogeneic stem cell transplantation [15,16]. The breaking of tolerance to self-antigens with Treg depletion may be functionally analogous to the anti-tumor effect seen in allogeneic bone marrow transplantation, whose primary side-effect, graft-versus-host disease, is evidence that tolerance to normal self antigens has been modified.

The serological analysis of recombinant cDNA expression libraries (SEREX) constructed from patient tumor was established by Sahin and Tureci who demonstrated that this process identifies T-cell antigens as well as B-cell antigens [17,18]. SEREX continues to be employed in patient studies and has even proven to identify intracellular antigens targeted by the immune system [19]. The identification of the NY-ESO-1 antigen in patients by SEREX demonstrated that both MHC class II restricted epitopes and MHC class I-restricted (HLA-A2) epitopes, targets of cytotoxic T cell responses, could be identified with this technique [19]. We present a new means to identify immunogenic tumor antigens. In this report we employ serum from experimental animals that have been vaccinated in the context of anti-CD25 antibody treatment, as opposed to using sera from tumor-bearing animals, which would be the equivalent of using serum from newly diagnosed patients. The use of immune serum-SEREX has allowed us to identify new tumor-associated antigens in our neuroblastoma model. Notably, we demonstrate that one of the antigens identified by our immune-SEREX approach, the DEK oncoprotein, induces a T cell response as well as an antibody response. Translation of this concept in to clinical studies would require the used of post-treatment or even post-vaccination serum, as opposed to the initial samples commonly harvested at the time of diagnosis.

## Results

The modification of syngeneic tumor cell lines with immune co-stimulatory molecules can transform lethal tumor cell lines into effective vaccines. The immunization of A/J mice with a cell-based vaccine, AGN2a-CD80/CD86, has been demonstrated to induce a strong cellular immune response [8]. This immune response is enhanced when the vaccine is given in the context of anti-CD25 antibody (clone PC61) as a means to block and/or deplete T-regulatory cells. We sought to explore the specificity of the immunoglobulin response to this cell based vaccine, as a means to potentially uncover new tumor-associated antigens. To do so, we constructed a cDNA library using mRNA purified from the AGN2a cell line and expressed proteins encoded by tumor- derived mRNA in E. coli using the λZAPII phage system. To identify clones expressing antigenic epitopes recognized by IgG present in immune sera, a total of 2 × 10^5 ^clones were screened. Fifty-eight unique plaques were identified as expressing proteins that could be recognized by IgG antibody when serum from AGN2a-CD80/CD86+PC61 treated mice was used at a dilution of 1:250. When pooled serum (n = 5 for each group) from naïve mice, tumor-bearing mice, mice treated with PC61 alone, or mice treated with two weekly injections of the vaccine cell-line AGN2a-CD80/CD86 alone was used to screen the cDNA library 0, 0, 0, or 5 unique clones, respectively, were identified. Antibody-reactive plaques identified by serum from the AGN2a-CD80/CD86+PC61-treated mice were picked, used to re-infect E. coli, and once more serologically screened. A third round of plaque purification was not required, as it always resulted in uniform recognition of phage-expressed proteins. In vivo excision was performed with helper phage and phagemid DNA isolated. Each phagemid insert was DNA sequenced using both T7 and T3 primers, and this sequence was used to search on-line databases. The 58 clones identified by immune serum-SEREX screening encoded 25 individual proteins, as listed in Table 1. Most notable was the frequency with which DEK was detected. While all antigens listed in Table 1 were detected once from a single positive clone, the DEK antigen was detected 34 separate times. Careful inspection of the cDNA library clones demonstrates that most transcripts seem to have some role in neuronal differentiation, cell cycle control, or have previously been identified as transcripts that are over-expressed in other malignancies. The final 4 transcripts listed in Table 1 have no published or annotated functional analysis associated with them, beyond their naming and characterization using bioinformatic techniques. Primers were generated for each clone identified, and expression of each verified by PCR analysis of AGN2a-derived cDNA (not shown).

**Figure 1:**
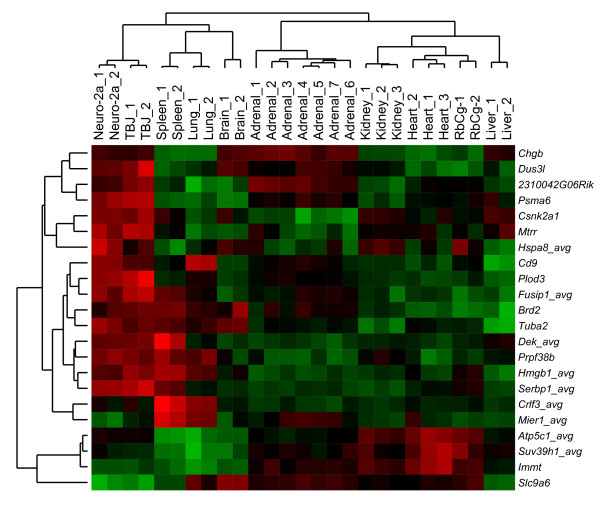
Gene Expression of DEK and other Neuroblastoma Tumor-Associated Antigens Identified by Immune Serum-SEREX. Expression of antigens in mouse neuroblastoma cell lines (the parental line for our vaccine, Neuro-2a, and TBJ) and normal mouse tissues are presented as a heatmap. A Total 22 of 25 SEREX identified genes listed in Table 1 are present in the mouse cDNA microarray chip and were used for the hierarchical clustering analysis. In the case of multiple clones representing the same gene, the average of expression ratios was used (labeled as GeneName_avg). The Pearson correlation matrix was used for clustering. Expression level of each gene was log2 transformed and z-score normalization was performed across samples for each gene. The expression was represented by pseudo-colors according to the scale shown on the bottom. A red color corresponds to up-regulation and a green color corresponds to down-regulation compared with the mean.

To further determine the significance of the SEREX identified transcripts, we performed cDNA microarray analysis to examine the expression level of these genes in mouse neuroblastoma cell lines Neuro-2A and TBJ or in mouse tissues. A total of 22 out of 25 genes were present in the array and were used for hierarchical clustering analysis, Figure 1. Importantly, the majority of the transcripts identified by SEREX are over-expressed in neuroblastoma cell lines, indicating the ability of immune serum-based SEREX analysis to identify over-expressed proteins using serum from vaccine-treated mice. Of note, DEK is indeed over-expressed in the neuroblastoma cell lines tested.

The cluster analysis displayed in Figure 1, identifies four different categories of neuroblastoma antigen expression profiles. In Cluster 1, transcripts are over-expressed in the mouse neuroblastoma cell lines in a tumor-restricted manner: Chgb, Dus31, 2310042G06Rik, Psma6 (proteasome subunit), Csnk2a1 (casein kinase), Mtrr (metabolic activation), and Hspa8 (heat shock protein). An interesting sub-group within this cluster includes Chgb, chromogrannin B, which is known to be associated with sympathetic neurons and which is also found in adrenal tissue. In Cluster 2 we find transcripts that are overexpressed in neurboastoma cells lines as well as spleen or lung: CD9 (tetraspanin, neuroblastoma marker), Plod3 (collagen biosynthesis), Fusip1 (neuronal differentiation), Brd2 (transcription), Tuba2 (tubulin alpha), DEK, Prpf38b, Hmgb1 (inflammatory mediator), and Serbp1 (serpine). Some of the proteins in this group, like CD9 and Hmgb1function as immune modulators. Cluster 3 (Crlf3 and Mier 1) and Cluster 4 (Atp5c1, Suv39h1, Immt, Slca6) are not over-expressed in neuroblastoma cells lines and seem unlikely to play a role in neuroblastoma. They may either be cross-reactive antigens or specific targets of auto-immune responses. We propose that each of the Cluster 1 and Cluster 2 transcripts should be explored with regard to tumorigenesis and immunogenicity.

**Figure 2:**
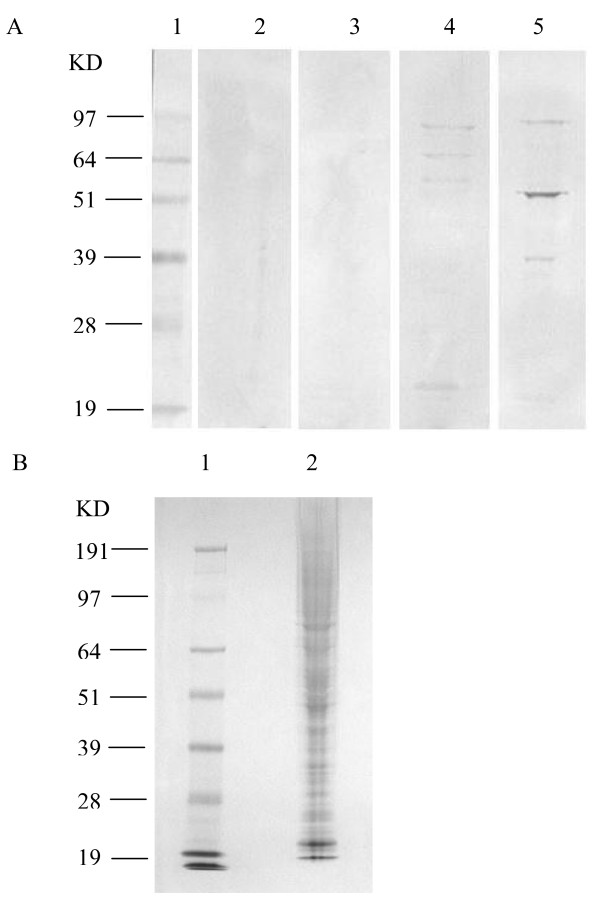
Western Blot Analysis of Whole Tumor Cell Lysates. (A) AGN2a cell lysate was prepared (as in Methods) and the indicated immune serum used at a dilution of 1:100. Lane 1, molecular weight marker (mw); Lane 2, naïve serum; Lane 3, serum from PC61-treated mice; Lane 4, serum from AGN2a-CD80/86 immunized mice; Lane 5, serum from AGN2a-CD80/86+PC61 (Treg-depleted)treated mice. (B) SDS-PAGE analysis showing, Lane 1, mw marker (mw) and, Lane 2, total AGN2a lysate (5 × 10^4 ^cell equivalents/well).

**Figure 3:**
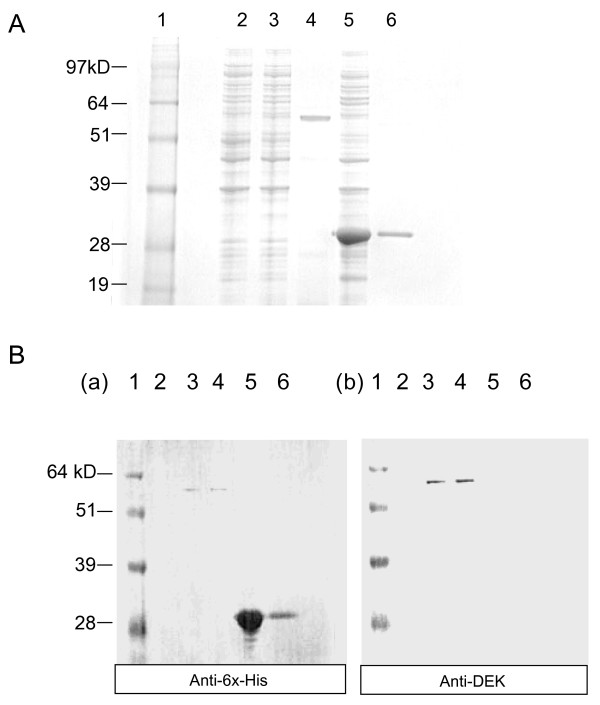
SDS-PAGE and Western Blot Analysis of Recombinant DEK. (**A**) Bacterial lysates and Ni-NTA column purified protein were diluted in reducing sample loading buffer, resolved by SDS-PAGE and stained with Coomassie blue. Lanes were loaded with 1) molecular weight (mw) marker, 2) control bacterial lysate, 10 μl, 3) pET-15b/DEK lysate, 10 μl, 4) 1.5 μg Ni-NTA column-purified DEK, 5) pET-15b/EGFP lysate, 10 μl, 6) 1.5 μg Ni-NTA column-purified EGFP. (**B**) Proteins were resolved by SDS-PAGE and then transferred to PVDF membrane using a Bis-Tris electrophoresis buffer system for western blot analysis. In blot (a) lanes contained: 1) mw marker, 2) control bacterial lysate, 3) pET-15b/DEK lysate, 10 μl, 4) 0.15 μg Ni-NTA column-purified DEK, 5) pET-15b/EGFP lysate, 10 μl, 6) 0.15 μg Ni-NTA column-purified EGFP. Blot (a) was probed with anti-6x-His antibody. For blot (b) a separate gel containing the same samples loaded in blot (a) was probed with anti-human DEK monoclonal antibody. Bound antibody was detected in both blots with alkaline phosphatase (AP)-conjugated rabbit anti-mouse IgG (H+L) secondary antibody, as described in Methods. Data is representative of more than three separate experiments.

Before we explored the specific immune response to DEK we sought to demonstrate that the immune response to a cell-based tumor vaccine is fundamentally altered by treatment with Treg-depleting antibody. The was first demonstrated qualitatively, whereupon western blotting of AGN2a cell lysates with serum from naïve, vaccinated (AGN2a-CD80/CD86), or vaccinated and Treg-depleted mice, different protein bands were recognized by the different sera, figure 2. Although prominent bands were seen with both the vaccine only group (figure 2A, lane 4), these differed from the prominent bands in the mice vaccinated in the context of Treg depletion (lane 5). To examine the differences in the response to a single protein we compared the antibody titer in an ELISA assay using recombinant DEK as the target and the same set of sera. Recombinant DEK protein was produced in the pET15b bacterial vector encoding an amino-terminal 6-histidine sequence to facilitate purification on a nickel affinity column. Enhanced green fluorescent protein (EGFP) was produced as a negative control. To obtain sufficient protein for immune function assays pET15b vectors were used to transform the BL21 (DE3) pLysS E. coli strain, protein expression induced with IPTG, and proteins containing the 6x-histidine tag purified from bacterial lysates using a Ni-NTA column. When columns were eluted with imidazole, distinct bands of 55 kD for DEK and 30 kD for EGFP were readily resolved by SDS-PAGE and Coomassie blue staining of the resolved proteins, Figure 3A.

**Figure 4:**
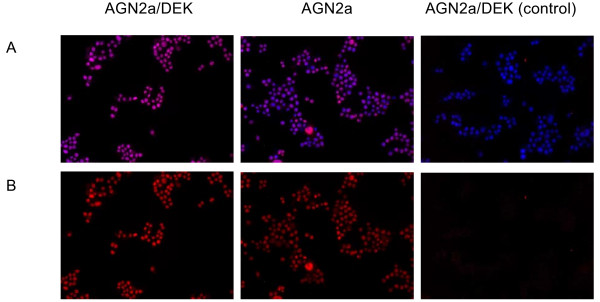
Immunofluorescence (IF) Analysis of DEK expression. AGN2a (column 2) or AGN2a/DEK (columns 1 and 3) were plated on glass chamber slides, fixed with 4% paraformaldehyde and visualized using a Nuance multispectral imaging system (Nuance 1P46, Cambridge Research and Instrumentation, Inc. Woburn, MA) mounted on a Zeiss Axio Imager Z1 microscope. Image files (200×) were captured and merged using the Axiovision 4.5 package (open program). For all cells, nuclei were stained blue with DAPI. Cells were stained with anti-human DEK antibody (1:100, columns 1 and 2) or similarly diluted isotype control antibody (column 3). Bound antibody was detected with goat anti-mouse IgG (H+L) conjugated to Alexa Fluor 555. Row (A) shows the merged DAPI and Alexa Fluor 555 image, and row (B) shows the staining of the Alexa Fluor 555 image alone. Data is representative of more than three separate experiments.

**Figure 5:**
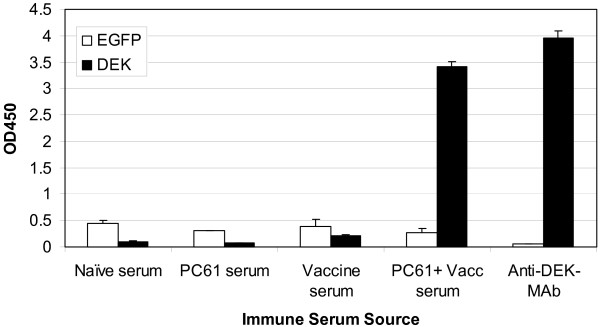
ELISA Analysis of Immune Sera for DEK Reactivity. 96-well plates were coated with recombinant DEK or EGFP (10 μg/ml) produced in bacterial vectors. Wells were blocked, and 100 μl of test serum (as described on the x-axis), diluted 1:100, was added to each well. Sera used for this assay was the same serum used for cDNA library screening, and was pooled from 5 immunized mice. Serum from naïve A/J mice, mice treated with PC61 alone, or AGN2a-CD80/CD86 vaccine alone was derived from parallel experimental groups used in the immunization protocol. Following incubation at 37°C for 1 hour, secondary rabbit anti-mouse IgG (H+L) was used to detect bound antibody, as detailed in Methods. Also included was anti-human DEK monoclonal antibody (Anti-DEK-MAb) used at 0.5 μg/ml. Error bars show standard deviations for triplicate sample wells in the same assay.

We then tested if purified DEK was recognized by anti-human DEK antibody and anti 6x-histidine antibody. Western blot analysis demonstrated that bacterially-expressed mouse DEK was recognized by anti-human DEK antibody and that anti-6x-histidine antibody recognized the histidine tag encoded by both recombinant proteins, Figure 3B. Having confirmed that the anti-human antibody recognized murine DEK, we looked at two cellular targets for the expression of DEK by immunofluorescence (IF). We analyzed both the wild-type/parental AGN2a cell line and AGN2a transfected with pcDNA3.1-Hygro/DEK (AGN2a/DEK), in order to induce over-expression of the DEK oncogene. Both AGN2a and AGN2a/DEK showed strong nuclear immunoreactivity by IF, with AGN2a/DEK showing more intense staining, Figure 4. While the expression of DEK was variable in AGN2a, strong expression was seen in every AGN2a/DEK transduced cell, as demonstrated by exact overlap of the DAPI and DEK signals. To further confirm that the phage-expressed DEK cDNA was a genuine immune target we tested serum from tumor-vaccinated mice for the presence of DEK-reactive immunoglobulin by ELISA. Using polystyrene plates coated with recombinant DEK, DEK-reactive IgG could be readily detected in serum from mice immunized with AGN2a-CD80/86 in the context of PC61 treatment, Figure 5. Anti-human DEK antibody was used as a positive control antibody in the ELISA. This assay also supports our hypothesis that vaccination in the context of Treg depletion alters the nature of the immune response to cell-based tumor vaccines. High titer DEK antibody production required the depletion of Treg, while vaccination with the cell based vaccine, AGN2a-CD80/CD86, did not produce anti-DEK IgG.

**Figure 6:**
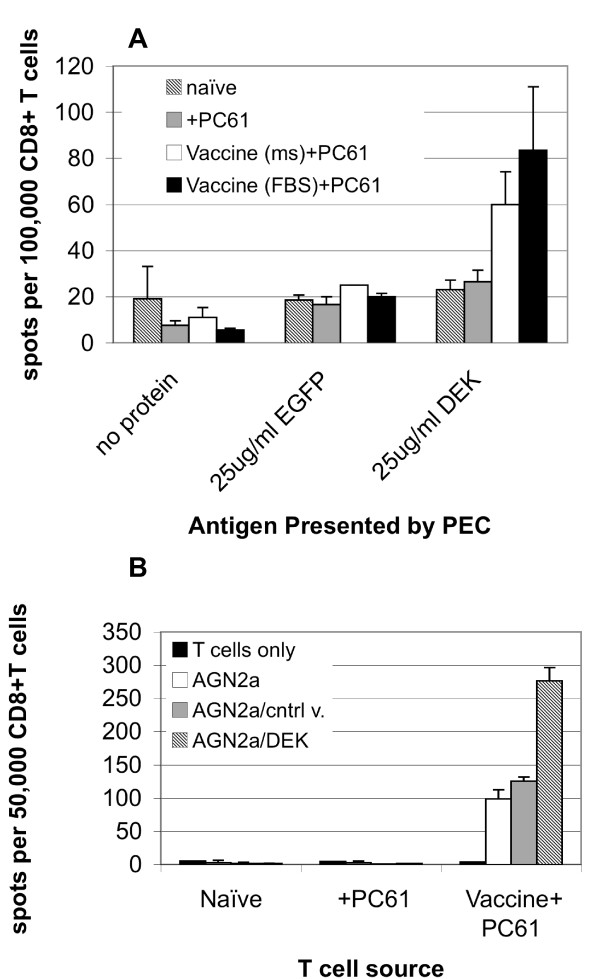
IFN-γ ELISPOT Analysis of CD8 T cells from Immunized Mice. CD8 T cells from immunized and control mice were purified by immunomagnetic sorting and co-incubated with the indicated APCs (protein-loaded PEC or tumor cells) for18 hours. **A**) 1 × 10^5 ^syngeneic PEC loaded with buffer (no protein), recombinant EGFP, or recombinant DEK were co-cultured with 1 × 10^5 ^CD8^+ ^T cells from the following groups of mice: naïve (stripes), PC61 treated (gray), PC61 treated plus AGN2a-CD80/86 vaccinated (cultured in normal mouse serum (ms)), (white), and PC61 treated plus AGN2a-CD80/86 vaccinated (cultured in FBS), (black). **B**) 5 × 10^4 ^CD8 T cells purified from naïve mice, PC61-treated mice, or AGN2a-CD80/86+PC61 treated mice were co-cultured with 1 × 10^4 ^tumor cells: AGN2a (white), AGN2a/pcDNA3.1 (gray, vector only control), AGN2a/DEK (stripes), or cultured alone (black, T cells only control). Data is representative of three separate experiments. Each experiment consisted of T cells purified and pooled from 5 mice. Error bars represent the standard deviation calculated from triplicate ELISPOT wells.

To investigate the CD8^+ ^T cell response against DEK in mice that had received a cell-based cancer vaccine, A/J mice were given two weekly subcutaneous (s.c.) injections of 2 × 10^6 ^irradiated AGN2a-CD80/CD86 cells cultured in DMEM supplemented with 2% mouse serum or 10% FBS, and Treg activity inhibited by the i.p. injection of clone PC61 anti-CD25 antibody three days prior to the first vaccination. Mouse serum-cultured vaccine cell lines were used in order to control for potential FBS reactivity, which has been reported in the analysis of CD4 T cell responses. AGN2a-CD80/86 was cultured in normal mouse serum at least two weeks prior to immunization studies. Splenocytes were collected 5 days after the second vaccination, depleted of RBCs, and CD8^+ ^T cells purified by immunomagnetic selection. T cell responses to DEK were detected in IFN-γ ELISPOT assays using two different sources of antigen-presenting cells. In the first assay, peritoneal exudate cells (PEC, which are primarily composed of macrophages) loaded with recombinant DEK protein were used. Harvested PEC were plated in ELISPOT plates, incubated with recombinant DEK or EGFP for 4 hours at 37°C, and then immune CD8^+ ^T cells added to the plates for an additional 18 hours. Control cultures containing T cells plus non-protein-loaded PEC, or T cells plus EGFP-loaded PEC, had similar low numbers of IFN-γ-producing cells, Figure 6A. In contrast, significant anti-DEK reactivity was seen in T cells from mice immunized with AGN2a-CD80/86 and treated with PC61, whether the vaccine cell line had been cultured in FBS or normal mouse serum (ms), Figure 6A.

The second source of antigen-presenting cells used to monitor anti-DEK responses was the AGN2a cell line we produced that over-expresses DEK (Figure 2). CD8^+ ^T cells were purified from naïve mice, mice treated only with PC61 (anti-CD25 antibody), or from mice that received both the AGN2a-CD80/86 vaccine and PC61. While naïve or PC61-treated mice did not show any IFN-γ ELISPOT activity in response to AGN2a, vaccinated mice showed relatively strong ELISPOT reactivity against both unmodified AGN2a tumor cells and heightened responses against the DEK-over-expressing cell line, Figure 6B. Taken together these assays demonstrate that anti-DEK CD8 T cell responses are induced by our vaccine+PC61 immunization protocol. This also validates that our serologic cDNA screening assay, based on the use of immune serum rather than tumor-onset serum, can identify important T cell epitopes.

## Discussion

SEREX analysis has been used to identify tumor-associated antigens in a number of malignancies. Primarily, patient serum has been used to screen tumor-derived cDNA libraries. The immunological rationale for the SEREX approach is that unique tumor antigens should induce an antibody response. However, the induction of tolerance to tumor antigens is now recognized to be a formidable obstacle to inducing anti-tumor immunity. It may well be that the antibody specificities present in tumor-bearing patients or animals represent antigens to which a tolerized, or non-tumoricidal immune response has been generated.

During our use of a pre-clinical model to test novel cell-based vaccines for neuroblastoma, we found that transfection of the AGN2a cell line with immune co-stimulatory molecules transforms the immunologically silent tumor into a powerful locus of immune activation. Moreover, immunization with a genetically modified tumor cell line, AGN2a-CD80/86, is even more effective when administered in the context of Treg blockade/depletion with an anti-CD25 mAb, PC61. In using PC61 it is also possible to deplete activated effectors, as all T cells express CD25 upon stimulation. Although we have not directly explored effects on antibody production, a single round of PC61 treatment prior to two weekly injections with a cell-based vaccine is superior to depletion three days prior to each of the weekly vaccinations when testing for cell-mediated anti-tumor immunity (Johnson, B.D., et al., 2007. *CD25+ Regulatory T Cell Inhibition Enhances Vaccine-induced Immunity to Neuroblastoma*, J. Immunother., in press). Based on this finding we used a single round of PC61 treatment in the studies presented here.

We have used serum from mice immunized twice weekly in the context of Treg-depletion to carry out a SEREX analysis, reasoning that unique neuroblastoma antigens may be uncovered in vaccinated, as opposed to tumor-bearing, animals. When an AGN2a cDNA library we prepared was screened for the ability to express IgG-reactive antigens, a number of transformation-associated proteins were identified, Table 1. These antigens were over-expressed in two other murine neuroblastoma cell lines as well, when compared to normal tissues, Figure 1, demonstrating that immunization in the context of Treg inhibition can identify unique and potentially important transcripts. What was most striking about our data was the abundant number of "hits" that were generated against the DEK oncogene. DEK was indeed over-expressed in neuroblastoma cell lines, Figure 1, and was recognized by immune serum in ELISAs, Figure 5. Our SEREX analysis also identified some antigens that were not over-expressed in neuroblastoma, yet these antigens still induced an antibody response. This response may either be due to the generation of cross-reactive antibody, or the antigens may be targets of an induced autoimmune response. It is also possible that point mutations in these antigens may have generated an immune response to them. Direct sequence analysis of these proteins will be required to confirm or refute this possibility and will be explored in future studies.

The combination of Treg depletion and vaccination may also induce CD8 effector cells. Recent descriptions of auto-reactive CD4 T cells that have expanded in vitro upon Treg depletion from healthy individuals including those specific for NY-ESO-1, tyrosinase, and GAD65 (a type 1 diabetes-associated autoantigen) support this hypothesis and suggest that Treg modulation may be essential for inducing anti-tumor immunity [20,21].

The identification of DEK as a tumor-associated antigen in murine neuroblastoma cell lines is fascinating due to the expression of DEK in an expanding number of human cancers. Both DEK and E2F3 have been identified as over-expressed transcripts due to chromosome 6p gains in retinoblastoma, a common pediatric malignancy [22]. Genomic gains of oncogenes like DEK are likely to be selected for because they confer a growth advantage to the malignancy, and 6p gains have been described in a number of malignancies including bladder and gastroesophageal cancer, osteosarcoma, and melanoma [23-26]. DEK was first identified as part of a fusion protein with the CAN/NUP 214 nucleoprotein in an acute myeloid leukemia, AML, sub-type through an "in-frame" translocation of chromosome 6 and 9 [27]. Biochemically, DEK is known to regulate transcription and contains a DNA binding domain, several phosphorylation sites, and a SAF-Box (scaffold attachment factor). DNA binding is dependent on phosphorylation by casein kinase 2 (CK2) which appears to regulate its transcriptional regulatory function [28-30]. Of note, we also identified CK2 by immune serum-SEREX, Table 1. Most recently DEK was demonstrated to reside in the spliceosome, and that upon phosphorylation, DEK associates with U2AF, enforcing 3' splice site discrimination, preventing U2AF from binding to pyridine tracts not followed by AG sequence [31]. The association with DEK over-expression or inactivation with human disease may be related to alterations in splice-site recognition and intron removal [32]. Evidence that DEK regulates key genomic responses to DNA damage was demonstrated by the ability of a partial fragment of DEK, isolated by a cDNA library screen, to complement an ataxia-telangiectasia phenotype in vitro [33]. In our immune-SEREX screen we identified partial transcripts as well, generated from an internal ATG sequence, that were recognized by immune sera (not shown). The association of DEK with cellular transformation has left little doubt of its oncogenic potential. DEK has been shown to inhibit senescence in cells infected with high-risk papillomavirus and to associate with the latency protein LANA-1 expressed by KSHV (Kaposi's sarcoma-associated herpesvirus) [34,35]. In the AML subset containing the t(6;9)(p23;q34) chromosomal abnormality, monitoring of the DEK-CAN transcript by RT-PCR shows an exact correlation to therapeutic outcome [36].

DEK is a nuclear protein, and antibody responses to DEK have been proposed to be indicators of autoimmune disease. However, in a report by Dong et al., it was proposed that anti-DEK antibodies are not a marker for any specific disease, but a marker for a subset of autoimmunity associated with IFN-γ production [37]. This is fascinating, as the breaking of tolerance to a self/tumor-associated antigen, as demonstrated by an IFN-γ mediated immune response, is a key characteristic of generating Th1 immunity. Further evidence for the ability to generate an immune response specific for DEK was seen when a human CD4 T cell clone specific to the DEK-CAN fusion protein produced IFN-γ upon co-culture with dendritic cells loaded with either apoptotic or necrotic t(6;9) leukemia cells [38]. Another potential mechanism for the induction of an immune response to self-proteins is the production of a truncated transcript by the tumor itself. Immune responses to a truncated HER-2/neu protein are far greater than to native protein and have become a new focus for vaccine development [39]. Although full-length recombinant DEK was used throughout our studies, the isolation of a partial transcript from the AGN2a cDNA library leaves this a possibility in our in vivo vaccine studies.

SEREX-based analysis of human cancers is entering a new phase of development. Studies from the laboratory of Dr. L. Old are beginning to describe how in different clinical situations, it is the SEREX-identified antibody targets that define discrete sets of over-expressed protein antigens that predict tumor pathophysiology. Notably, their current hypothesis is that SEREX specifically identifies tumor/self antigens that are recognized by CD4^+^CD25^+ ^regulatory T cells, and that without induction of an inflammatory immune response that includes CTL activity, immunization with SEREX-identified antigens alone may actually enhance tumor progression [40,41]. Our approach avoids this concern, in that tumor antigens we identify in the context of cell-based vaccination and Treg inhibition occur in a physiological environment where protective cytolytic T cell responses are being induced by the tumor cell-based vaccine [8]. We anticipate testing our hypothesis in human subjects immediately after the initial course of chemotherapy, where tumor antigen has been newly loaded in to the antigen-presenting cells, or following specific immunotherapeutic trials.

## Conclusion

We have presented a new rationale for tumor antigen identification using serum from mice vaccinated in the context of altered tolerance, by blocking the function of Treg cells. This procedure, which was developed for inducing strong T cell responses, also generated a strong serological response. Few if any antigens were identified in tumor-bearing, or Treg-blocked only animals. However, vaccination in the context of Treg depletion produced serum from which we were able to generate a candidate tumor antigen list, and we demonstrate that for one of these antigens, the DEK oncogene, a strong T cell response is also induced. A number of these antigens are associated with tumorigenicity and should be explored in their own right. We conclude that tolerance mechanisms are operative in tumor-bearing animals, and that these may block effective tumor antigen identification by serological screening of cDNA libraries (SEREX). To extrapolate these findings to human disease, serology-based antigen discovery should be carried out not with onset or diagnostic serum (which is most commonly banked), but with serum derived from treated patients in which antigen loading in to the immune system is optimal and in which Treg effects may be minimized.

## Methods

### Mice and tumor cell lines

A/J mice, 6–8 weeks of age, were purchased from Jackson Laboratories (Bar Harbor, ME). Mice were housed in the Medical College of Wisconsin Biomedical Resource Center (AALAC accredited) and all protocols were approved by the MCW Institutional Animal Care and Use Committee. AGN2a, an aggressive clone of Neuro2a, was derived from successive in vivo passage, and AGN2a transfectants that permanently express CD80 and CD86 (AGN2a-CD80/86) have been previously described [8].

### Generation of immune serum

A/J mice were given two weekly subcutaneous (s.c.) injections of 2 × 10^6 ^irradiated AGN2a-CD80/86 cells. For blockade/depletion of T-regulatory cells, mice received 500 μg of bioreactor generated (Integra CL 1000, Chur, Switzerland) anti-CD25 monoclonal antibody (mAb), clone PC61, by intraperitoneal (i.p.) injection 3 days prior to the first vaccination. Blood was collected 5 days after the second vaccination, incubated at 37°C for 30 min, centrifuged at 800 × g for 10 min, and then stored at -80°C.

### Construction of cDNA expression libraries

Total RNA was isolated from AGN2a using Trizol (Invitrogen) according to the manufacturer's protocol and mRNA purified using the Oligotex mRNA Kit (Qiagen). 5 μg of purified poly-A mRNA was used to construct a cDNA library using the ZAP Express cDNA Synthesis Kit and ZAP Express cDNA Gigapack III Gold Cloning Kit (Stratagene, Inc., La Jolla, CA). Three libraries based on size fractionation of packaged cDNA inserts were created. cDNA fragments were cloned into the λZAPII Express Vector (Stratagene), packaged into phage particles, and used to transfect E. coli, resulting in at least 1.25 × 10^5 ^primary recombinants per library. We screened the library with the highest titer (2 × 10^7 ^pfu/ml) and the most representative insert size species for mammalian mRNA (600–2500 bp as determined by PAGE).

### Immunoscreening of the AGN2a cDNA library

Proteins encoded by the cDNA expression library were probed with sera from AGN2a-CD80/86+PC61 vaccinated mice (pooled from 5 mice). Recombinant phage at a concentration of 5,000 pfu per plate (150 mm^2^) were amplified for 4 hr at 42°C until plaques were visible and then transferred to nitrocellulose membranes pre-wetted with 10 mM IPTG (Invitrogen) for an additional 3.5 hr at 37°C. Membranes were then washed 3 times with TBST (20 mM Tris-Cl, 150 mM NaCl, 0.05% Tween 20, pH7.5), blocked with 1% bovine serum albumin (BSA, Sigma A3803) in TBS, and then incubated with a 1:250 dilution of immune serum, which had been pre-adsorbed with E. coli phage lysate following the manufacturer's protocol (Stratagene). Bound antibody was detected by incubation with alkaline phosphatase-conjugated rabbit anti-mouse IgG (H+L) (Abcam) and visualized by staining with 4-nitro blue tetrazolium chloride/5-bromo-4-chloro-3-indoyl-phosphate (NBT/BCIP, picoBLUE Immunoscreening Kit, Stratagene). Positive clones were subcloned and re-screened as above. In vivo excision was carried out with plaques that proved positive upon secondary screening in order to generate the pBK-CMV phagemid containing the cloned insert. Phagemid was isolated with QIAprep columns (QIAGEN) and the size of the cDNA insert analyzed by XhoI/EcoRI restriction digest. Inserts were sequenced using T7 and T3 primers by automated DNA sequencing (ABI 3100, MCW Protein and Nucleic Acid Facility). To verify that phagemid-encoded cDNA was expressed by AGN2a, newly generated cDNA (with Olido-dT and SuperScript III, as above) was screened by PCR (34 cycles at an annealing temperature of 55°C) using primers specific for each gene. Primers were designed on-line using Primer3 [42].

### Expression of DEK protein

Full-length DEK (GeneBank, BC055451) was cloned from AGN2a cDNA using the following primers: (fwd) 5'-GGAATTCCATATGCCGGGTCCCAGGGAAGAG, (rev) 5'-CGGGATCCTCAAGAAATTAGCTCTTTTACAG. A small portion of non-translated 5' sequence was included to produce an in-frame product for protein expression and to overcome the repetitive GC-rich region just prior to the start codon. Primers used to amplify and clone EGFP were: (fwd) 5'-CATATGGTGAGCAAGGGCGAG, (rev) 5'-GGATCCGCTTTACTTGTACAGCT. NdeI and BamHI restricted PCR fragments were ligated into pET-15b (Novagen, Madison, WI) and recombinant plasmid insert sequence (pET-15/DEK and pET-15/EGFP) was verified by DNA sequencing. Plasmids were transformed into E. coli strain BL21 (DE3) and gene expression induced with 0.8 mM isopropyl-β-D-thiogalactopyranoside (IPTG, Invitrogen). Prokaryotically-expressed proteins were purified using a Ni-NTA Purification System (Invitrogen) and analyzed by SDS-PAGE and western blotting. Bacterial lysates were lysed in reducing loading buffer (NuPAGE system, Invitrogen), proteins resolved by SDS-PAGE, and the proteins transferred to PVDF membranes (Invitrolon, 0.45 μm, Invitrogen) using a NuPAGE Bis-Tris electrophoresis system (Invitrogen). Blots were probed with anti-human DEK (BD Biosciences) and anti-His antibody (Serotec, Raleigh, MC) at a 1:1000 dilution, followed by alkaline phosphatase conjugated rabbit anti-mouse IgG (H+L) (Abcam) at a 1:2500 dilution. NBT/BCIP was used for AP detection (picoBLUE Immunoscreening Kit, Stratagene).

To produce stable transfected cell lines, AGN2a was transfected by electroporation with the pcDNA3.1-Hygro vector (Invitrogen) encoding DEK. Transfected cells were selected by culture in 400 μg/ml hygromycin (Invitrogen), cloned by limiting dilution, and subclones selected for uniform DEK expression by immunofluorescent staining, as follows: 1 × 10^5 ^cells were plated overnight in glass chamber slides (Nalge, Nunc International), washed 2× with PBS, and fixed with 4% paraformaldehyde (Sigma) for 15 minutes at room temperature. The slides were rinsed in PBS, blocked with 10% normal goat serum, and then incubated overnight at 4°C with mouse anti-human DEK (1:100, BD Biosciences) or isotype control (IgG1, 1:100, BD Biosciences). Slides were then rinsed in PBS and then incubated for 1 hour with Alexa Fluor 555-conjugated goat anti-mouse IgG (H+L), (1:1000, Invitrogen). After rinsing in PBS, the cells were incubated for 5 minutes with 0.3 mM DAPI (Invitrogen) at room temperature. Slides were mounted with Vectashield (Vector Laboratories) and microscopically inspected.

### Western blot analysis

AGN2a cells were washed twice with PBS, resuspended in PBS at 5 × 10^6^/ml, and diluted in three volumes 4× sample buffer (NuPAGE LDS Sample Buffer, Invitrogen, Inc.). (A) Lane 1, molecular weight (mw) marker and lanes 2 through 5, boiled AGN2a cell lysate (5 × 10^4 ^cell/well). After resolution of proteins by SDS-PAGE (12%, NuPAGE gel system, Invitrogen, Inc.), proteins were transferred to PVDF membrane using a Bis-Tris electrophoresis buffer system and transferred to Invitrolon PVDF membrane (as above), then cut into strips. Lanes were blocked by incubation for 1 hr at room temperature in 5% non-fat dry milk and 1% BSA in Tris-buffered saline, pH 7.5, rinsed and then incubated in: Lane 2, serum from naïve mice; Lane 3, serum from PC61 treated mice; Lane 4, serum from AGN2a-CD80/86 immunized mice; and Lane 5, serum from AGN2a-CD80/86 immunized +PC61 treated mice, diluted 1:100 in blocking buffer. Bound antibody was detected with biotin-conjugated goat anti-mouse IgG (Biotin-SP-conjugated AffiniPure Goat Anti-Mouse IgG, Fcγ Fragment Specific, Jackson ImmunoResearch Laboratories, Inc., West Grove, PA) diluted 1:1000 in blocking buffer, washed, then incubated with AP labeled-ExtraAvidin (Extravidin-Alkaline Phosphatase, Sigma) and detected with picoBLUE as above. Data is representative of more than three separate experiments. (B) Lane 1, molecular weight marker; Lane 2, Coomassie blue stain of proteins resolved by SDS-PAGE of the AGN2a cell lysate.

### Vaccination and immune assays

A/J mice were immunized by subcutaneous (s.c.) injection of 2 × 10^6 ^irradiated (5000 rad) AGN2a-CD80/86 cells cultured in DMEM supplemented with 2% mouse serum (Equitech-Bio) or 10% FBS (Gemini Bio-Products) in the context of PC61 treatment (as above). Five days following the second of two weekly vaccinations, splenocytes were collected, depleted of red cells, and CD8^+ ^T cells purified using the CD8a (Ly-2) Microbead kit (Miltenyi Biotech) on an AutoMACS device (Miltenyi Biotech). ELISPOT analysis to enumerate CD8^+ ^IFN-γ-producing cells was carried out using the BD ELISPOT mouse IFN-γ Set and 96-well PVDF membrane plates (Millipore, Bedford, MA) according to the manufacturer's protocols. In some assays, peritoneal exudate cells (PEC) were used as antigen-presenting cells. 1 × 10^5 ^PEC from naïve A/J mice were placed in ELISPOT wells and loaded with protein by incubating in 100 μl media containing recombinant DEK or EGFP at 25 μg/ml for 4 hours at 37°C. 1 × 10^5 ^CD8^+ ^T cells (in 100 μl) were added to each well for 18 hr to test for antigen recognition. For direct recognition of tumor cells, 1 × 10^4 ^neuroblastoma cells (AN2a or AGN2a/DEK) were incubated with 5 × 10^4 ^CD8^+ ^T cells. Spots were counted using an automated reader (Immunospot 3, C.T.L., Ltd., Cleveland, OH).

DEK-specific IgG was detected by coating 96-well plates (EIA/RIA, Costar, Corning, NY) with bacterially-expressed DEK or EGFP (1 μg per well) in carbonate buffer (45.3 mM NaHCO_3_, 18.2 mM Na_2_CO_3_, pH 9.6). Diluted sera was added to blocked wells and detected with rabbit anti-mouse IgG (H+L) labeled with alkaline phosphatase (Abcam) and developed with NNBT/BCIP (Stratagene).

#### Microarray analysis

The normal mouse tissues (brain, heart, lung, rib cage, spleen, liver and kidney) and tumors established using TBJ and Neuro-2a murine neuroblastoma cell lines were used for microarray analysis. For each tissue or tumor, at least two samples were used. Total RNA was purified using a combination of Trizol extraction followed by Qiagen column purification [43]. We utilized mouse cDNA microarray chips consisting of 19940 probes representing 13,958 non-redundant genes in a cDNA microarray experiment carried out precisely as previously described [43]. NIH3T3 RNA was used as reference in all hybridizations. To investigate the expression of SEREX identified transcripts in different mouse tissues and neuroblastoma cell lines, we used UGRepAcc to match the SEREX identified genes and the genes existing in cDNA array, 22 of 25 SEREX-identified genes having matches. In the case of multiple clones representing the same gene, the average of expression ratio was used. The data were log2 transformed and z-score normalization was performed across samples for each gene. Hierarchical clustering analysis was performed using the Pearson distance as the distance measure.

## Authors' contributions

JZ was responsible for experimental procedures and carried out cloning, plaque screening, T cell assays. MEK carried out experiments and developed protein purification protocols. QC analyzed and complied gene expression profiling data. JW carried out experiments and assisted in analyzing data. JK established normal tissue and neuroblastoma expression profiling databases. BJ designed and analyzed all immune assays in animals. RO was responsible for the overall design and execution of the experimental program. All authors have read and approved the final manuscript.

## Figures and Tables

**Table 1:** Neuroblastoma antigens identified by immune serum and SEREX.

**Identity of cDNA SEREX hit**	**Accession**	**Characteristics**	**Ref**
**DEK**	BC055451	p53 interaction, blocks apoptosis DNA binding/chromosomal organization, Proofreading of splicing Fused to ***can ***in leukemia	Wise-Draper '06 Waldman '04 Soares '06 Garcon '05
**Hspa8, heat shock protein 8 (Hsc70)**	NM_031165	Promotes cell-cycle progression (w/cyclin D1) Binds/regulates dbl proto-oncogene	Diehl '03 Kauppinen '05
**Fusip1, FUS interacting protein (Nssr, TASR)**	BC0832082	Neuronal Differentiation, modulates neuronal mRNA splicing Over-expressed in liposarcoma and leukemia	Liu '04 Fushimi '05 Clinton '02
**Hmgb1, high mobility group box-1**	BC008565	Inflammatory mediator DNA binding, architecture regulation	Andreason '02 Stott '06
**Atp5c1, ATP synthase (mitochondrial F1 complex, gamma polypeptide 1)**	BC010700	ATP biosynthesis, upregulated in STI571-resistant leukemia	Hofmann '02
**Sfrs5, splicing factor, arginine/serine-rich (SRp40, HRS)**	BC085267	Splicing factor, Akt substrate Apoptosis resistance	Patel '05 Tidd '03
**Prpf38b, pre-mRNA processing factor (PRP38)**	BC050900	snRNP, required for spliceosome activity	Xie '98
**Tuba1, tubulin, alpha 1**	BC085256	Upregulated in carcinoma	Qi '05
**Chn1, chimerin 1**	BC010825	Neurotrophin target	Mizuno '04
**Csnk2a1, casein kinase II, alpha 1 polypeptide**	BC060742	Phosphorylates/activates NFkappaB	Chantome '04
**Mkks, McKusick-Kaufman syndrome protein**	NM_021527	Chaperonin, required for cytokenesis	Kim '05
**Mier1, mesoderm induction early response 1 (Mi-er1, er1)**	AK129405	Fibroblast growth factor induced, HDAC1 interaction, transcription factor	Ding '03 Post '05
**MTTR, 5-methyltetrahydrofolate-homocysteine methyltransferase reductase**	BC025942	Mutations seen in neural tube defects Influences ALL susceptibility	Wilson '99 Gemmati '04
**Psma6, proteasome subunit, alpha type 6**	NM_011968	Upregulated in hepatocellular ca.	Cui '06
**CD9**	NM_007657	Cell surface tetraspannin, neuronal differentiation and neuroblastoma marker	Ishibashi '04 Komada '00
**Chromogranin B, Chgb (secretogranin I)**	NM_007694	Present in neuroblastoma secretory vesicles	Goodall '97
**Brd2, bromodomain containing 2 (NAT, Rnf3, Ring3, Fsrg1)**	NM_001025387	Binds to KSHV, LANA-1 protein Lymphomagenesis	Viejo-Borbolla '05 Greenwald '04
**Crlf3, cytokine receptor-like factor 3**	AL591113	Upregulated in skin cancer	Dang '06
**Plod3, procollagen-lysine, 2-oxoglutarate 5-dioxygenase 3 (LH3)**	NM_011962	Collagen biosynthesis Upregulated in chemo resistant ca.	Rautavuoma '04 Cleator '05
**Immt, inner membrane protein mitochondrial (HMP; P87; P89; P87/89)**	AK038129	Energy metabolism	John '05
**Suv39h2, suppressor of variegation 3–9 homolog 2**	AF149205	Epigenetic regulation of telomere length	Garcia-Cao '04
**Serbp1, serpine1 mRNA binding protein**	BC006030	inferred function (by Gene Ontology)	
**Dus3l, dihydrouridine synthase 3-like**	AY040840	inferred function (by Gene Ontology)	
**Slc9a6, Solute carrier family 9 (Na+/H+ exchanger)**	AK028350	inferred function (by Gene Ontology)	
**2310042G06Rik**	BC026968	Riken cDNA, predicted protein	

The table lists the name (identity) of the recombinant plaques in the AGN2a cDNA library that expressed IgG-reactive protein using serum from mice immunized with AGN2a-CD80/86 in the context of PC61 treatment three days prior to first vaccine. Each transcript was identified a single time, with the exception of DEK, as detailed in Results. The table also lists the accession number, relevant characteristics of the identified protein, and the references that initially reported these functions. All information is derived from publicly available databases including Entrez Gene, National Center for Biotechnology Information, National Library of Medicine, U.S. National Institutes of Health.
